# Consequences of the introduction of a new protein during the recovery period of a chronic intestinal inflammation

**DOI:** 10.1186/1939-4551-8-S1-A261

**Published:** 2015-04-08

**Authors:** Airton Silva, Sylvia Campos, Monique Bitteti, Karla Pepe, Gerlinde Teixeira

**Affiliations:** 1 Universidade Federal Fluminense, Brazil

## Introduction

New food introduced to an inflamed gut may result in sensitization to this food. AIM Evaluate the time required for recovery after chronic inflammatory intestinal process for the development of oral tolerance to new proteins.

## Methods and results

Male C57BL/6 mice (n=30) were immunized with 100μg of peanut protein. Half received a 30-day raw-peanut-challenge-diet (CD) (inflamed-I) while the other received mouse chow (controls-C)(Teixeira 2008). They were further divided and received sweetened OVA (new protein) (egg white diluted in distilled H_2_O, 1:5 v/v+5% sucralose) orally for 7 days, on day 0 (I1 C1), 10 (I2 C2) or 20 (I3 C3) post CD. We assessed body weight, food intake, antibodies and T cell phenotype of mesenteric lymph nodes (MLN) and spleen (SPN). ANOVA with Tukey post-test was performed. Statistical significance considered p<0.05. This study was approved by UFF’s Ethical Committee (CEUA #00147-09). During CD groups I2 (-1.94±2.2) and I3 (-1.44±1.8) but not I1 (0.15±0.6) showed significant weight loss compared to C. The C groups (37.50±4.6) ingested significantly more kcals compared to I1 (31.20±2.6), I2 (30.78±2.5) and I3 (30.84±3.4). OVA consumption was significantly lower in I1 (3.21±1.0) compared to I2 (6.53±1.19), I3 (6.82±2.3) and C (7.5±1.7). In the MLN we observed a significant increase of CD8^+^T cells of I1 (29.49±4.1) and I2 (31.72±4.0) compared to I3 (21.53±3.6) and C (25.65±5.4) and CD8^+^CD25^+^T I1 (0.43±0.2) when compared to I2 (0.32±0.0), I3 (0.24±0.1) and C (0.30±0.1) with no significant difference in CD4^+^T cells (I 37.34±5.7 and C 38.35±5.1) and CD4^+^CD25^+^ T cells (I 6.86±1.5 and C 7.00±1.9). In SPN, we observed significantly increase of CD4^+^T cells in I1 (38.67±2.5) compared to I2 (25.93±3.48), I3 (24.48±5.9) and C (26.25±12.2) which was not accompanied by the increase of CD4^+^CD25^+^Foxp3^+^T cells. We observed a significant increase in the CD8^+^CD25^+^T cells in I1 (0.25±0.1) compared to I2 (0.11±0.0), I3 (0.1+0.0) and C (0.1±0.1). The I-1 showed gut inflammation, increased intraepithelial leukocytes and destruction/flatning of the villi and drop of goblet cells. The I-2 and 3 were similar to C.

## Conclusions

Aversion to novel proteins by animals with gut inflammation may be a protective mechanism to the induction of multiple allergies.

